# PKC Regulates YAP Expression through Alternative Splicing of YAP 3′UTR Pre-mRNA by hnRNP F

**DOI:** 10.3390/ijms22020694

**Published:** 2021-01-12

**Authors:** Wing-Keung Chu, Li-Man Hung, Chun-Wei Hou, Jan-Kan Chen

**Affiliations:** 1 Healthy Aging Research Center, Chang Gung University, Taoyuan 33302, Taiwan; d9701303@cgu.edu.tw (W.-K.C.); lisahung@mail.cgu.edu.tw (L.-M.H.); romeomonkey@msn.com (C.-W.H.); 2 Department and Graduate Institute of Biomedical Sciences, College of Medicine, Chang Gung University, Taoyuan 33302, Taiwan; 3 Kidney Research Center, Chang Gung Memorial Hospital, Linkou 333, Taiwan; 4 Department of Physiology, College of Medicine, Chang Gung University, Taoyuan 33302, Taiwan

**Keywords:** alternative splicing, hnRNP F, PKC, 3′UTR

## Abstract

The Yes-associated protein (YAP) is a transcriptional co-activator that plays critical roles in organ development and tumorigenesis, and is verified to be inhibited by the Hippo signaling pathway. In the present study, we show that the YAP 3′UTR is alternatively spliced to generate a novel 950 bp 3′UTR mRNA from the full length 3′UTR region (3483 bp) in human cancer cells. The ratio of full length 3′UTR YAP mRNA to alternatively spliced 3′UTR YAP mRNA is up-regulated by exposure of the cells to PKC inhibitor chelerythrine chloride. Further study using luciferase reporter assay showed that the expression of the alternatively spliced 3′UTR mRNA is much lower compared with the full length 3′UTR mRNA, suggesting that alternatively spliced 3′UTR YAP mRNA may have a shorter half-life than full length 3′UTR mRNA. Interestingly, PKC represses YAP 3′UTR–mediated mRNA stability is dependent on a splicing factor, hnRNP F. Activation of PKC induces nuclear translocation of cytosolic hnRNP F. Ectopic expression of hnRNP F enhances YAP 3′UTR splicing. Our results suggest that hnRNP F regulates YAP 3′UTR-mediated mRNA stability in an alternative splicing-dependent manner, and PKC regulated YAP expression is dependent on nuclear translocation of hnRNP F in human cancer cell lines.

## 1. Introduction

Yes-associated protein (YAP) and transcriptional co-activator with PDZ-binding motif (TAZ) are the main downstream effectors of the mammalian Hippo pathway, which exert a crucial role in controlling the tissue and organ development and tumorigenesis. Previous studies reported that enhanced YAP expression and nuclear translocation have been detected in many human cancers, including colon, liver, lung, ovary and prostate cancers [1,2,3,4]. Overexpression of YAP was shown to increase cell proliferation and mediate cellular transformation in vitro [5,6]. Recently, two meta-analysis indicated that high YAP expression is correlated with poor outcome and reduced survival in several human cancers [7,8]. Therefore, YAP may be suitable for use as a predictive biomarker for cancer diagnosis and as a potential target for designing anti-cancer drugs. Furthermore, previous studies have demonstrated the correlation between diabetes and cancer [9,10,11]. Because of the significant increase in the prevalence of these two diseases [12,13,14], it is vital to understand the molecular basis governing the functional interplays of diabetes and cancer, as well as the possible role of YAP in these diseases. YAP protein expression was shown to be elevated in the kidney cortical tissues of both type 1 (streptozotocin mimics) and type 2 (*db/db*) mouse diabetic models. In patients with type 2 diabetes and nephropathy, YAP and TEAD were highly expressed in kidney tissues, and were correlated with systolic blood pressure, blood urea nitrogen, creatinine, stages of the diabetic nephropathy, serum albumin and glomerular filtration rate, suggesting that YAP played a significant role in the renal damage in type 2 diabetes [13,15].

Approximately 95% of multi-exon genes in human can generate multiple transcripts, expands proteome complexity during cell differentiation and tissue maturation. The alternative splicing may be critical in supporting accurate development to form specific tissues. However, many alternative splicing occurs in the 3′ untranslated regions (UTRs), and has no effect on the polypeptide sequence but instead may regulate mRNA stability, translation and localization. For instance, Rox8, a RNA-binding protein inhibiting cell proliferation and tissue growth in Drosophila development, promotes yki (the *Drosophila* ortholog of YAP) mRNA decay via binding to its binding site in yki 3′UTR, suggesting that 3′UTRs play crucial roles in *yki*/*YAP* mRNA metabolism during *Drosophila* development [16]. Furthermore, TIAR, the human ortholog of Rox8, is also able to degrade *yki* mRNA when introduced into *Drosophila* and destabilizes *YAP* mRNA in human cells, indicating the retention of a conserved regulatory function in *yki*/*YAP* mRNA stability.”

Heterogeneous nuclear ribonucleoprotein F (hnRNP F) is 415-amino-acids long RNA-binding protein belonging to the hnRNP family that plays roles in cytoplasmic trafficking of mRNAs, posttranscriptional modification and mRNA stabilization. hnRNPs contains sequence-specific RNA-binding activity and their activities are conferred by RNA-recognition motifs [17]. hnRNP F has three repeats of quasi RNA recognition motifs (quasi-RRMs), specifically recognizes poly(G) sequences and exhibits binding preferences of GGGA or DGGGD (where D is U, G, or A nucleotide) motifs [18,19]. It has been reported that hnRNP F regulates alternative splicing of various genes [20,21]. In *Drosophila*, the hnRNP F homolog, Glorund was also shown to recognizes poly(G) sequences to regulate alternative splicing, implying that alternative splicing events are conserved in both human and *Drosophila*. Overexpression of hnRNP F enhanced *Ace-2* gene transcription, avoids TGF-β1 inhibition of *Ace-2* gene transcription, prevents hypertension and kidney injury in diabetes [22]. Deficiency of hnRNP F in mice was shown to elevate systolic blood pressure and prompt glycosuria through regulating renal *Agt* and *Sglt2* expression, respectively [23]. Furthermore, hnRNP F enhanced *sirtuin-1* gene expression, which defends against oxidative stress and diminishes diabetic nephropathy in mice [24].

Although YAP has been shown to play role in the diabetic nephropathy and in tumor formation, the molecular mechanisms of the regulation of *YAP* gene expression remained unknown. Our previous report showed that YAP 3′-untranslated region (3′UTR) plays a role in regulating mRNA stability. HnRNP F negatively regulated YAP expression by decreasing YAP mRNA stability, and the effect was through interaction with a poly(G) sequences (G-tract) in YAP 3′UTR [25]. In the present study, we found that the YAP 3′UTR was alternatively spliced to generate a 950 bp 3′UTR mRNA from the full length 3′UTR region (3483 bp). Alternatively spliced 3′UTR YAP mRNA exhibited a shorter half-life than full length 3′UTR mRNA. hnRNP F enhanced YAP 3′UTR splicing and repressed YAP 3′UTR-mediated mRNA stability in an alternative splicing-dependent manner. Our results suggested that hnRNP F regulates YAP expression through alternative splicing of YAP 3′UTR pre-mRNA. Inhibition of the YAP expression might have the effect in preventing tumorigenesis and delaying progression of the diabetic nephropathy.

## 2. Results

### 2.1. Generation of an Alternatively Spliced YAP 3′UTR in Human Cancer Cells

**Figure 1:**
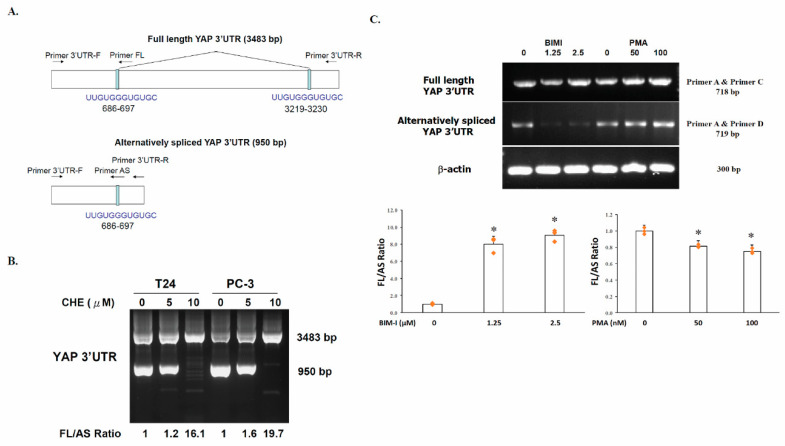
Inhibition of PKC activity reduces the YAP alternatively spliced YAP 3′UTR mRNA. (**A**). Schematic presentation of the full-length (top) and alternatively spliced (bottom) isoforms of the YAP 3′UTR. Arrows indicate primer positions for PCR amplification of YAP 3′UTR isoforms. The alternatively spliced YAP 3′UTR is generated by the splice deletion of base pairs between 697 and 3218. The locations of short direct repeat sequences are indicated. (**B**). T24 and PC-3 cells were incubated with chelerythrine chloride at the indicated concentrations. Twenty-four hours after incubation, the total RNA was harvested and the RT-PCR assay was performed. YAP 3′UTR s were amplified by RT-PCR. The bands were scanned with a densitometer, and the ratios of the full length to alternatively spliced 3′UTR were calculated. *β-actin* gene was used as an internal control for RNA quality and loading. (**C**). PC-3 cells were treated with increasing concentrations (0, 1.25, and 2.5 µM) of bisindolylmaleimides I (BIM-I) or PMA (0, 50, and 100 nM) for 24 h (*n* = 3). Cells were lysed, and total RNA was used for RT-PCR using specific primers for full-length YAP 3′UTR, alternatively spliced YAP 3′UTR and β-actin. Significance was tested using one-way analysis of variance (ANOVA) with Holm–Sidak method post hoc test, where * *p* < 0.05 compared to the control.

*YAP1* gene spans approximately 122.94 kb with 7 exons. The open reading frame of the coding region is 1364 bp. The human YAP 3′UTR is 3483 bp in length. In the present study, we found that the 3′UTR of the *YAP* gene was alternatively spliced (AS) to form a 950 bp 3′UTR mRNA with a deletion of from nucleotides 698 to 3218 in human cancer cells. Using a specific primer pairs (Primer 3′UTR -F and Primer 3′UTR -R) that spans either side of the 3′UTR of the *YAP* gene (Figure 1A), we found that the alternatively spliced form of YAP mRNA was expressed in PC-3 (human prostate adenocarcinoma cancer cells) and T24 cells (human bladder carcinoma cells) (Figure 1B). The full-length YAP 3′UTR was found to cotain two 12-base G-tracts (UUGUGGGUGUGC) direct repeat (Figure 1A, top). While the alternatively spliced YAP 3′UTR (Figure 1A, bottom; 950 bp) contained only one of the two 12-base repeat. It is likely that these shortened direct repeat sequences may be involved in the generation of deletions in the 3′UTR of the *YAP* gene.

### 2.2. The Switch of YAP 3′UTR between Full Length 3′UTR and Alternatively Spliced 3′UTR Is Regulated by PKC

To examine whether the switch in splicing was regulated by PKC, the PC-3 and T24 cells were incubated with chelerythrine chloride, a broad-spectrum PKC inhibitor, at 5 µM and 10 µM, respectively, followed by RNA extraction and RT-PCR using primers that detect both full length (FL) and alternatively spliced (AS) isoforms. Treatment with chelerythrine chloride increased the relative abundance of the full length (Figure 1A, top) form over the alternatively spliced form (Figure 1A, bottom) from 1.2-fold to 16.1-fold in T24 cells (Figure 1B) and from 1.6-fold to 19.7-fold in PC-3 cells.

A semi-quantitative RT-PCR was used to analyze alternatively spliced products. By designing primers for constitutively retained nucleotide sequences, it is possible to simultaneously amplify isoforms that contains with or without the target nucleotides (Primer 3′UTR -F and Primer 3′UTR -R) (Figure 1A,B). Alternatively, RT-PCR can be used to specifically amplify the full-length YAP 3′UTR by using one primer in the constitutively retained sequence and an opposing primer in an alternatively retained portion (Primer 3′UTR-F and Primer FL). To detect the alternatively spliced form, a boundary-spanning primer (Primer AS) for the sequence encompassing the junction with the primer in constitutive retained sequence can be used (Primer 3′UTR -F) (Figure 1A). The effect of PKC on YAP 3′UTR splicing was further confirmed by semi-quantitative RT-PCR using isoform specific primers. Consistently, alternatively spliced YAP 3′UTR (AS) level was found to be dose-dependently repressed by PKC inhibitor bisindolylmaleimides I (BIM-I) treatment in PC-3 cells. Bisindolylmaleimide I, a more selective PKC inhibitor, showed high selectivity for PKCα, β1, β2, γ, δ, and ε isozymes, were used to show the splicing is specifically mediated by PKC activity. The alternatively spliced YAP 3′UTR level was decreased by approximately 83% and 92%, respectively, in cells treated with 1.25 µM and 2.5 µM of BIM-I (Figure 1C). When the ratio of the FL to AS in the untreated cells, was set as 1, the ratio of BIM-I treated cells were calculated to be 8.0 and 9.0, respectively, at a drug concentration of 1.25 μM and 2.5 μM. In contrast, treatment of the cells with the phorbol ester (PMA), a classical and novel PKCs, at 50 nM and 100 nM resulted in the elevation of alternatively spliced YAP 3′UTR level by approximately 45% and 84%, respectively, compared to that of the untreated cell.

### 2.3. YAP Is Upregulated by PKC Inhibitors in Human Cancer Cells

**Figure 2:**
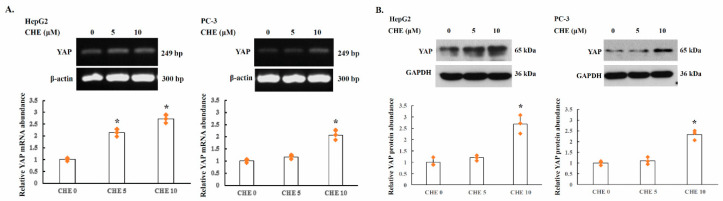
YAP expression is enhanced by PKC inhibitor in HepG2 cells and PC-3 cells. ((**A**). top). Cells were treated with PKC inhibitor chelerythrine chloride at the indicated concentrations for 24 h and the expression of YAP and β-actin transcripts was assessed by semi-quantitative RT-PCR assay as described in the Materials and Methods (*n* = 3). Amplified PCR products were resolved on a 1% agarose gel. ((**A**). bottom) Quantitative real-time PCR was used to quantify the expression levels of YAP. Values were normalized using β2-microglobulin mRNA. (**B**). For Western blot analysis, cells were incubated with chelerythrine chloride at the indicated concentrations. Cell lysates were prepared 24 h after treatment, and were subjected to Western blot analysis with anti-YAP, and anti-GAPDH antibodies (*n* = 3). Significance was tested using one-way analysis of variance (ANOVA) with Holm–Sidak method post hoc test, where * *p* < 0.05 compared to the control.

The effect of PKC activity on YAP expression was further studied in HepG2 and PC-3 cells. The cells were incubated overnight with chelerythrine chloride and the YAP levels were examined by RT-PCR and Western blot analysis. For semi-quantitative RT-PCR and quantitative real-time RT-PCR analysis, the primers were designed to amplify open reading frame of *YAP* gene, as described in the Materials and Methods. As shown in Figure 2, YAP mRNA level was increased by approximately 125% and 171%, respectively, in HepG2 cells treated with 5 µM and 10 µM of chelerythrine chloride. Consistently, the YAP protein levels in HepG2 cells treated with chelerythrine chloride at 5 µM and 10 µM were also elevated by approximately 20% and 169%, respectively, compared to that of the untreated cell. In PC-3 cells, YAP mRNA level was also elevated approximately 16% and 105% in 5 µM and 10 µM chelerythrine chloride treated cells, compared to that of the untreated cells. The YAP protein levels in PC-3 cells were also elevated by approximately 10% and 132%, respectively.

The results showed that YAP the expression levels of YAP alternatively spliced 3′UTR mRNA is inversely with the levels of full length 3′UTR mRNA in PC-3 cells. Thus, a molecular switching of YAP mRNA between full length 3′UTR and alternatively spliced 3′UTR in PC-3 cells is involved in the regulation of YAP protein expression.

### 2.4. Alternative Splicing of YAP 3′UTR Contributes to Its Stability

**Figure 3:**
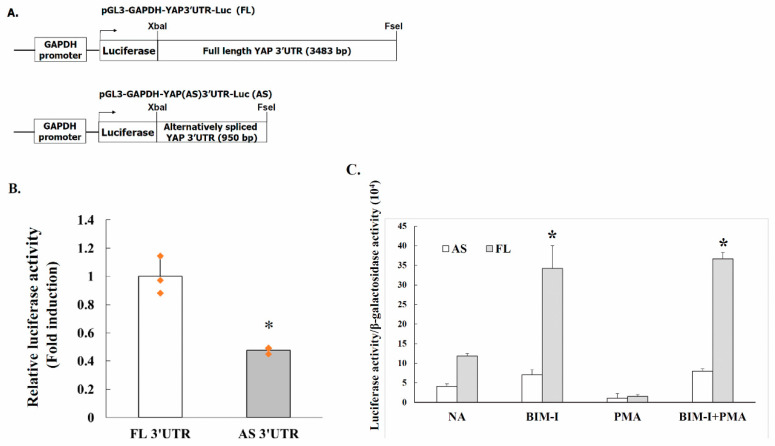
Increased PKC activity decreases YAP 3′UTR mRNA stability (**A**). Schematic presentation of constitutive luciferase reporter constructs (not drawn to scale). The 3′UTR full length and alternatively spliced 3′UTR of the YAP were cloned into the XbaI and FseI sites downstream to the firefly luciferase gene in the sense orientation. Transcription (bent arrow) was driven by the GAPDH promoter upstream to the luciferase reporter gene. (**B**). Luciferase construct containing YAP full length (FL) and alternatively spliced (AS) 3′UTR were transfected into PC-3 cells. The cells were also cotransfected with pSV-β-galactosidase vector, and the β-galactosidase activity was used to normalize the luciferase activity (*n* = 3). Asterisks indicate a significant difference compared to the YAP full length 3′UTR. * denotes *p* < 0.05 (*t*-test). (**C**). The PC-3 cells were transfected with pGL3-GAPDH-YAP-3′UTR-Luc or pGL3-GAPDH-YAP(AS)-3′UTR-Luc. Luciferase activity was assayed after the treatment of the cells with 2.5 µM BIM-I or 50 nM PMA for 16 h (*n* = 3). “BIM-I + PMA” represent that the cells were preincubated with BIM-I for 30 min before treatment with PMA. Asterisks indicate a significant difference between the alternatively spliced 3′UTR and the YAP full length 3′UTR. * denotes *p* < 0.05 (*t*-test). NA: not added.

The regulation of mRNA stability is an central control point in determining the protein abundance [26]. We used a constitutive luciferase reporter to determine if FL 3′UTR and AS 3′UTR YAP mRNA exhibit different cellular stability. Firefly luciferase constructs containing YAP full length or alternatively spliced 3′UTR s were generated by PCR amplification using primers containing the XbaI and FseI sites. PCR-amplified products with the XbaI and FseI sites were cloned in pGL3-GAPDH-Luc, containing a constitutive GAPDH promoter and the YAP 3′UTR s were placed immediately after the luciferase gene at the 3′ region (Figure 3A). The resulting luciferase reporter constructs were individually co-transfected with the pSV-β-Galactosidase vector to PC-3 cells and the cells were harvested 24 h after transfection. The luciferase activity of the transfected cells was then measured. As shown in Figure 3B, luciferase activity is significantly decreased in cells transfected with the alternatively spliced 3′UTR construct compared with the full length 3′UTR construct. Strongly suggesting that YAP alternatively spliced 3′UTR confers instability.

### 2.5. PKC Activity Decreases YAP 3′UTR–Mediated mRNA Stability

We then asked whether PKC affected YAP 3′UTR-mediated mRNA stability, PC-3 cells were transfected with luciferase reporter constructs pGL3-GAPDH-YAP(FL)3′UTR-Luc or pGL3-GAPDH-YAP(AS)3′UTR-Luc, respectively, and the reporter activity was examined. As shown in Figure 3C, treatment of cells with 2.5 µM of PKC inhibitor BIM-I enhanced full-length YAP 3′UTR (pGL3-GAPDH-YAP(FL) 3′UTR-Luc) reporter activity up to 191%, suggesting that PKC may facilitate YAP 3′UTR mRNA stability. In contrast, similar treatment led to a mere 73% increase of the alternatively spliced YAP 3′UTR luciferase reporter (pGL3-GAPDH-YAP(AS) 3′UTR-Luc) activity.

The possible effects of PKC activator phorbol 12-myristate 13-acetate (PMA) was also examined. The cells transfected with full-length or alternatively spliced YAP 3′UTR luciferase reporter were exposed to PMA with or without pretreatment with PKC inhibitor BIM-I. As shown in Figure 3C, PMA exerted a repressive effect on reporter activity. The inhibitory effect of PMA was reversed by pretreatment of the cells with PKC inhibitor BIM-I, suggesting that decreased PKC pathway activity promotes mRNA stability. Taken together, the results suggested that elevated PKC activity downregulates YAP expression by a post-transcriptional mechanism.

### 2.6. PKC Represses YAP 3′UTR–Mediated mRNA Stability Is Dependent on hnRNP F

**Figure 4:**
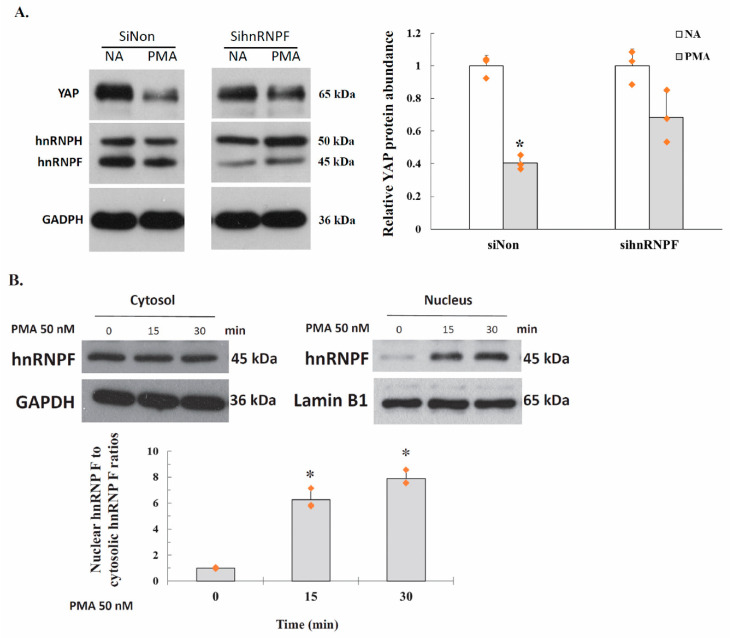
Knockdown of hnRNP F prevents the down-regulation of YAP expression by activated PKC and PKC activation enhances increased hnRNP F nuclear translocation. (**A**). PC-3 cells were transfected with siNon or sihnRNPF (40 nM each) for 24 h (*n* = 3). The cells were then incubated with 50 nM PMA. Cell lysates were prepared 24 h after treatment, and were subjected to Western blot analysis with anti-YAP, anti-hnRNP F/H and anti-GAPDH antibodies. Significance is tested using two-tailed Student’s *t*-test, where * denotes *p* < 0.05 compared to the same siRNA transfected cells not treated with PMA. (**B**). PC-3 cells were treated with 50 nM PMA for 24 h (*n* = 3). Cell lysates were prepared, and fractionated into cytosolic and nuclear fractions. The fractions were analyzed by immunoblot using anti-hnRNP F. anti-GAPDH and anti-Lamin B1 were used as cytosolic and nuclear loading controls. Fold changes are represented as relative values of band densities normalized to the control. Significance was tested using one-way analysis of variance (ANOVA) with Holm–Sidak method post hoc test, where * *p* < 0.05 compared to the untreated cells.

An important question is how PKC enhance 3′UTR alternative splicing. In a previous study, we showed the YAP3′UTR plays a role in regulating mRNA stability and YAP expression 20. A hnRNP F binding site, a G-tract RNA sequence (5′-UUUGUGGGUGUGCA-3′) was identified in the YAP 3′UTR at 685 to 698. We found that hnRNP F downregulates YAP expression via interaction with the G-tract. Interestingly, our study also showed hnRNP U also exhibit repressive effects on YAP expression as that of hnRNPF, however, hnRNP U was not recruited to the G-tract. In the present study, we therefore examined how hnRNP F affects YAP 3′UTR alternative splicing, PC-3 cells were transfected with siRNA against either control or hnRNP F for 24 h. Cells were then treatment with 50 nM PMA for another 24 h, and cell lysates were prepared and used for Western blot analysis. As shown in Figure 4A, the YAP protein levels was downregulated by approximately 59% in PMA treated, control siRNA transfected cells, compared to that of the untreated cells. In contrast, YAP expression was decreased by approximately 32% in PMA treated, hnRNP F siRNA transfected cells, compared to that of the untreated cells. The results suggested that elevated PKC activity suppresses YAP expression is at least, partially dependent on hnRNPF.

### 2.7. Activation of PKC Induces Nuclear Translocation of Cytosolic hnRNP F

The effect of PKC activity on hnRNP F nuclear translocation were analyzed. PC-3 cells were incubated with 50 nM PMA for 0, 15 or 30 min. Cells were fractionated into cytosolic and nuclear fractions. The fractions were analyzed by immunoblotting using anti-hnRNP F. anti-GAPDH and anti-Lamin B1 antibodies. In untreated cells, hnRNP F were localized predominantly to the cytosol fraction (Figure 4B). In contrast, in PMA treated cells, hnRNP F was localized mostly to the nucleus with a concomitant decrease in the cytosol. When the ratio of the Nuclear hnRNP F to cytosolic hnRNP F in the untreated cells, was set as 1, the ratio of PMA treated cells were calculated to be 6.3 and 7.9, respectively, at 15 min and 30 min after PMA exposure. Briefly, activation of PKC by PMA significantly induced the translocation of hnRNP F protein from cytosol to the nucleus.

### 2.8. HnRNP F Enhances YAP3′UTR Splicing

**Figure 5:**
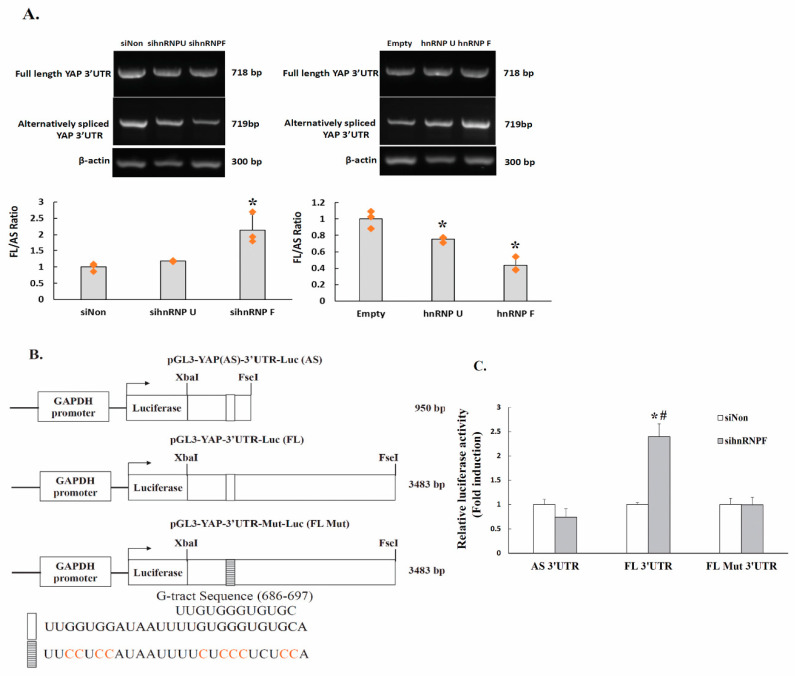
HnRNP F induces YAP 3′UTR splicing and the effect of hnRNP F on YAP 3′UTR-mediated mRNA stability is alternative splicing dependent. (**A**). PC-3 cells were transfected with siNon, sihnRNPU, sihnRNPF (40 nM each) (left panel), or were transfected with empty vector, pFRT-TO-HIS-FLAG-HA-hnRNPU or p3X-FLAG-hnRNPF at 0.5 μg/mL (right panel). Twenty-four h after incubation, total RNA was isolated and the semi-quantitative RT-PCR assay was performed (*n* = 3). PCR fragments were amplified by RT-PCR using primers as described in the Figure 1C, to specifically amplify the full-length YAP 3′UTR and alternatively spliced YAP 3′UTR. *β-actin* gene was used as an internal control for RNA quality and loading. Significance was tested using one-way analysis of variance (ANOVA) with Holm–Sidak method post hoc test, where * *p* < 0.05 compared to the control. (**B**). Schematic diagram of the luciferase reporter used in assay. Another G-tract located in full-length YAP 3′UTR at 3219 to 3230 was not shown in this diagram. (**C**). PC-3 cells were transfected with 0.5 μg of pGL3-YAP(AS)-3′UTR-Luc, pGL3-YAP-3′UTR-Luc or pGL3-YAP-3′UTR-Mut-Luc along with 40 nM siRNA against non-silencing control (siNon) or hnRNP F (sihnRNPF) for 24 h (*n* = 3). The cells were also cotransfected with a pSV-β-galactosidase vector, and the β-galactosidase activity was used to normalize the luciferase activity. Luciferase activity is presented as the fold induction relative to the control. Data are means ± s.d from triplicate analysis. Significance is tested using ONE-WAY ANOVA with Holm-Sidak method post hoc test, where * denotes *p* < 0.05 compared to the siNon transfected cells; where # denotes *p* < 0.05 compared to the cells cotransfected with pGL3-YAP(AS)-3′UTR-Luc and same siRNA.

To further ascertain the regulatory roles of hnRNPF in PKC mediated YAP mRNA splicing, the PC-3 cells were transfected with siRNA against either hnRNP U or hnRNP F, and the mRNA levels of the alternatively spliced YAP 3′UTR were compared As shown in Figure 5A, left panel, the alternatively spliced YAP 3′UTR mRNA levels were down-regulated by approximately 4% and 29%, respectively, in cells transfected with siRNA against hnRNP U and hnRNP F, compared to that of the non-silencing siRNA transfected cells. Thus, knockdown of hnRNP U or hnRNP F elevated the relative abundance of the full length form over the alternatively spliced form by approximately 17% or 114%. The effects were further confirmed by ectopically expressed hnRNP F or hnRNPU. The cells transfected with either hnRNP F (p3X-FLAG-hnRNPF) or hnRNP U (pFRT-TO-HIS-FLAG-HA-hnRNPU) expressing vectors and the abundance of the alternatively spliced YAP 3′UTR mRNA was evaluated. The results showed that ectopically expressed hnRNP U and hnRNP F elevated alternatively spliced YAP 3′UTR (Figure 5A, right panel), by approximately 57% and 150%, respectively, compared to that of the control cells. Thus, the relative abundance of the full length form over the alternatively spliced form was decreased by approximately 25% and 57%, respectively, in cells transfected with p3X-FLAG-hnRNPF and pFRT-TO-HIS-FLAG-HA-hnRNPU.

### 2.9. The Regulation of YAP 3′UTR-Mediated mRNA Stability by hnRNPF Is Alternative Splicing-Dependent

Our previous study showed that hnRNP F represses YAP 3′UTR-mediated mRNA stability via a G-tract in YAP 3′UTR 20. In the present study, we further suggest that hnRNP F elevates the ratio of alternatively spliced YAP 3′UTR to full length form. We thus further investigate whether the stability of the two YAP 3′UTR isoforms may have different respond to hnRNP F. The PC-3 cells were cotransfected with luciferase reporters and siRNA against either control or hnRNP F and the reporter activity was determined. Furthermore, to functionally test the roles of the G-tract in hnRNPF effects, the 685 to 698 region of the full-length YAP 3′UTR was mutated (pGL3-YAP-Mut-3′UTR-Luc) (Figure 5B) and the reporter activities were then assayed. As shown in Figure 5C, cells cotransfected with pGL3-YAP-3′UTR-Luc and hnRNP F siRNA resulted in a 125% increase of the reporter activity. In contrast, cotransfection of pGL3-YAP(AS)-3′UTR-Luc with hnRNP F siRNA led to a 26% decrease of the luciferase reporter activity. Intriguingly, mutation of the G-tract attenuated the stimulated effect of hnRNP F-siRNA on full-length pGL3-YAP-3′UTR-Luc reporter activity. The results suggested that hnRNP F promotes alternative splicing of the full-length YAP-3′UTR leading to increased levels of the alternatively spliced YAP 3′UTR, and the G-tract is critical in the alternative splicing-dependent YAP-mRNA stability. Taken together, the results suggested that hnRNPF interacts with G-tract to promotes alternative splicing to generate alternatively spliced YAP 3′UTR mRNA, which is less stable compared to that of the full length YAP 3′UTR mRNA.

## 3. Discussion

We previously reported that YAP 3′UTR plays a role in regulating mRNA stability and YAP expression. YAP mRNA stability is negatively regulated by hnRNP F in human cancer cells [25]. A G-tract was identified in the YAP 3′UTR at 685 to 698. hnRNP F binds to a G-tract sequence (at 685-698) in YAP 3′UTR mRNA and is involved in the hnRNP F-regulated YAP mRNA stability. In the present study, we found that the YAP 3′UTR was alternatively spliced to generate a 950 bp 3′UTR mRNA from the full length 3′UTR region (3483 bp) in human cancer cells. The ratio of full length 3′UTR YAP mRNA to alternatively spliced 3′UTR YAP mRNA was up-regulated by exposure of the cells to PKC inhibitor chelerythrine chloride (Figure 1). YAP protein expression was inversely correlated with the level of YAP alternatively spliced 3′UTR mRNA and proportionally correlated with full length 3′UTR mRNA in PC-3 cells (Figure 2). Thus, a molecular switching of YAP mRNA between full length 3′UTR and alternatively spliced 3′UTR in PC-3 cells is probably involved in the regulation of YAP protein expression. Further study using luciferase reporter assay showed that the expression of the alternatively spliced 3′UTR is much lower compared with the full length 3′UTR mRNA, suggesting that alternatively spliced 3′UTR YAP mRNA may have a shorter half-life than the full length 3′UTR mRNA (Figure 3B). We also showed that inhibition of PKC activity increases YAP 3′UTR–mediated mRNA stability via alternative splicing of YAP 3′UTR, and thus contributes to YAP mRNA stability (Figure 3C). Interestingly, PKC repressed YAP 3′UTR–mediated mRNA stability is dependent on hnRNP F (Figure 4A). Activation of PKC induces nuclear translocation of the cytosolic hnRNP F (Figure 4B). Thus, it appeared that a direct link exists between PKC activation and hnRNP F translocation to the nucleus. hnRNPs are present in the cell nucleus during post-transcriptional modification of the newly synthesized pre-mRNA, leading to alternative splicing and ectopic expression of hnRNP F enhances YAP 3′UTR splicing (Figure 5A). Our results suggested that hnRNP F regulates YAP 3′UTR-mediated mRNA stability in an alternative splicing-dependent manner (Figure 5C). We also suggested that PKC regulate YAP expression through alternative splicing of YAP 3′UTR pre-mRNA and the PKC effect is hnRNP F dependent in human cancer cell lines. Since the G-tract is involved in alternative splicing, it is therefore, curious to see if the G-tract sequence is also present in the 3′UTR of the core components of Hippo pathway, such as TAZ, MST1 and LATS1/2. The 3′UTR of YAP, TAZ, MST1 and LATS1/2 were aligned using CLUSTAL O (1.2.4) multiple sequence alignment. The result has been attached as a supplementary Data S1. Consistently, the results showed that the G-tract sequence is located in YAP 3′UTR. However, the sequence is not present in TAZ, MST1 and LATS1/2. Activation of the EGF receptor may regulate cell proliferation, apoptosis, differentiation and is essential for development, and dysregulation of these pathways can promote tumorigenesis. Previous studies have demonstrated that EGFR is activated in experimental models of diabetic kidney injury and in cultured renal cells exposed to high glucose [13,27,28]. In diabetic nephropathy, EGFR-PI3K-Akt-CREB activated signaling pathway may elevated the YAP expression [13]. Recently, YAP/TAZ has been reported to promote fibroblast activation and renal fibrosis [29,30]. Connective tissue growth factor (CTGF/CCN2), a pro-inflammatory and profibrotic factor, has been shown to markedly elevated in fibrotic conditions, and strongly suggested to be involved in the development and progression of kidney injury in diabetes [13,31]. In renal epithelial cells, CTGF were recognized as downstream target genes of YAP activation in response to high glucose [13]. Moreover, YAP/TAZ are mechanoregulators that bind to Smad transcription factors, the central mediators of TGF-β elicited profibrotic responses in fibroblasts, suggesting that TGF-β may induce renal fibrosis in a YAP/TAZ- and Smad2/3-dependent manner [29].

The control of cellular protein level is achieved essentially by regulating mRNA levels. The turnover of an mRNA is finely regulated by cis-acting elements located in the 3′UTR, and their associated trans-acting factors. The RNA binding proteins (RBPs), function as trans-acting factors recognizing cis-acting elements, may bind directly with RNA transcripts to regulate several mechanisms including alternative splicing, turnover, localization and translation in cells. In our previous study, we proposed that YAP 3′UTR plays a role in regulating YAP mRNA stability and YAP protein level in human cancer cells. hnRNP F likely destabilizes YAP mRNA by interacting with the G-tract of the YAP 3′UTR. One possible way that hnRNP F represses YAP expression is through the alternative splicing. Alternative splicing of pre-mRNAs is a vital mechanism in the gene expression, which is implicated in mRNA stability and translation. HnRNP F has been demonstrated to regulates alternative splicing of many mRNA precursor, such as Bcl-x. The quasi-RRMs of hnRNP F specifically recognizes poly(G) sequences, RNA sequences that are critical for splice site recognition [20]. Since hnRNP F are well known regulator of pre-mRNA processing, alternative splicing is one reasonable explanation for the hnRNP F-repressed YAP expression. Because the instability of the alternatively spliced YAP 3′UTR mRNA contributes to the decreased expression of YAP protein in human cancer cells. We assumed that the full length YAP 3′UTR mRNA contains crucial cis-elements that bind trans-acting factors that may confer stability, and when the region is spliced out the mRNA becomes unstable. In summary, the present study showed that hnRNP F plays a negative regulatory role on YAP expression through alternative splicing of YAP 3′UTR. Our finding suggested that hnRNP F may be important in preventing diabetic nephropathy and in controlling tumorigenesis.

## 4. Materials and Methods

### 4.1. Cell Line and Cell Culture

Human prostate adenocarcinoma cancer cells (PC-3), human urinary bladder carcinoma cells (T24) and human hepatocellular carcinoma cells (HepG2) were obtained from Bioresource Collection and Research Center, Taiwan. Cells were incubated in Dulbecco’s Modified Eagle’s Medium (DMEM) (Invitrogen, Carlsbad, CA, USA) supplemented with 10% Fetal bovine serum, 1 mM pyruvate, 2 mM L-glutamine at 37 °C in a humidified incubator with 5% CO_2_ and 95% air.

### 4.2. Reverse Transcription-PCR (RT-PCR) and RT-qPCR

Total RNA was extracted using TRIzol reagent (Invitrogen) according to the protocol recommended by the supplier and reverse-transcribed by Superscript III First-Strand Synthesis System (Invitrogen). The PCR was carried out for 25–35 cycles at 95 °C for 1 min, 55 °C for 1 min, 72 °C for 1 min in 2× GoTaq^®^ Green Master Mix (Promega, Madison, WI, USA). RT-qPCR was used to quantify the YAP mRNA levels. Aliquots (0.1 μL) of the cDNA samples were performed in iQ SYBR Green Supermix (Bio-Rad, Hercules, CA, USA) using Roche LightCycler 480 System. The program consists of 95 °C for 3 min and 40 cycles of 95 °C for 15 sec and 60 °C for 1 min. Data were averaged and relative quantification was determined by the ΔΔCt method. The results were normalized to β2-microglobulin mRNA. The synthetic oligonucleotide primer sequences used in this section are provided in Supplemental Table S1A.

### 4.3. Construction of YAP 3′UTR Containing Luciferase Constructs

To generate the luciferase reporter constructs expressing firefly-luciferase in a YAP mRNA 3′UTR dependent manner, the pGL3-GAPDH-Luc, containing a constitutive GAPDH promoter, was created by replacement of the vector specific luciferase 3′UTR with the human YAP 3′UTR [25]. The 3′UTR fragment of *YAP* gene was amplified using the primer pairs containing a XbaI site and a FseI site for the subsequent cloning reactions. The primer sequences are provided in Supplemental Table S1A. PCR products containing full length YAP 3′UTR and alternatively spliced YAP 3′UTR were cloned into pGL3-GAPDH-Luc to generate pGL3-YAP-3′UTR-Luc and pGL3-YAP(AS)3′UTR-Luc reporter.

### 4.4. Site-Directed Mutagenesis of the YAP 3′UTR-Luciferase Reporter Vectors

To construct a YAP 3′UTR containing the mutated G-tract, site-directed mutagenesis of the YAP 3′UTR-luciferase plasmids was performed using PCR. The PCR reaction mixture contained 1 μg pGL3-YAP-3′UTR-Luc containing the YAP 3′UTR, 12.5 μL 2× Phusion High-Fidelity DNA Polymerase (Finnzymes Oy, Espoo, Finland) and 0.1 μg of each primer. The primers used were: 5′-TGCCACATACTCTAATATAGATTTTCCTCCATAATTTTCTCCCTCTCCATTTTGTTCTGTTTTGTTGGGTTT-3′(sense) and 5′-AAACCCAACAAAACAGAACAAAATGGAGAGGGAGAAAATTATGGAGGAAAATCTATATAGAGTATGTGGCA-3′(antisense). PCR was carried out at 94 °C for 30 s, 55 °C for 60 s, and 68 °C for 8 min for 10 cycles. The methylated parental DNA templates were then digested with 20 U DpnI (NEB, Schwalbach, Germany) at 37 °C for 8 h. The DNA fragment containing the desired mutation was transformed into competent E. coli DH5α cells. The presence of the desired mutation was verified by direct DNA sequencing [25].

### 4.5. Transient Transfection and Western Blot Analysis

The cells were incubated at 37 °C until 70% confluence and then transfected with lipofectamine 2000, Life Technologies, Inc.) with p3X-FLAG-hnRNPF [32], pFRT-TO-HIS-FLAG-HA-hnRNPU [33] or pCMV-Empty plasmid. The cells were seeded in 60 mm dishes until 70% confluence and then transfected with lipofectamine 2000 (Invitrogen) with p3X-FLAG-hnRNPF, pFRT-TO-HIS-FLAG-HA-hnRNPU or pCMV-Empty plasmid at room temperature for 20 min before adding to the cells. All sense siRNAs were purchased from MWG-Biotech (AG, Germany) The siRNA sequences are listed in Supplementary Table S1B. Transfection was performed using Lipofectamine 2000 (Invitrogen) for 24 h. After transfection, the cells were washed with twice pre-cold PBS and lysed in RIPA Lysis Buffer (Millipore, Bedford, MA, USA). Protein concentrations were quantified by the Bio-Rad protein assay kit (Bio-Rad). Protein lysates were resolved on a 10% SDS-polyacrylamide gel and electroblotted onto Immobilon-P PVDF membranes (Millipore). The membranes were blocked in TTBS containing 5% nonfat milk and incubated with diluted primary antibodies overnight at 4 °C. The membranes were then reacted with species-specific HRP-conjugated secondary antibodies (1:5000–10,000 in TTBS), and the immunoreactive protein bands were detected by Amersham ECL™ Prime Western Blotting Detection Reagent (Amersham, Piscataway, NJ, USA). Primary antibodies used including GAPDH (1:50,000; Millipore, MAB374, USA), Lamin B1 (1:10,000; Abcam, ab16048, Cambridge, UK) YAP (1:2000; Cell Signaling Technology, #4912, Beverly, MA, USA), and hnRNP F/H (1:1000; Abcam, ab10689, Cambridge, UK).

### 4.6. Luciferase Reporter Assay

Transient transfection of luciferase reporter plasmids was performed using Lipofectamine 2000 (Invitrogen), according to the manufacturer’s instructions. Cells were plated in 12-well tissue culture plates at 2 × 10^5^ per well for overnight. Nucleic acid-lipofectamine 2000 mixtures containing 0.5 μg of luciferase reporter vectors, 0.5 μg of pSV-β-galactosidase control vector (Promega) and 0.5 μg of expressing vectors or 40 nM siRNA were exposed to cells. After 24 h, cell lysates were harvested and assayed for the Luciferase activity assay (Promega), performed according to the protocol recommended by the supplier. To normalize the transfection efficiency, the same cell lysates were also assayed for β-galactosidase activity using the β-Galactosidase enzyme assay (Promega).

### 4.7. Statistical Analysis

The data were presented as mean values ± standard deviation from at least three independent experiments, each being performed in triplicate. To compare data between two groups, two-tailed Student’s t-test were performed. Data from different groups were compared using one-way ANOVA with Holm–Sidak method post hoc test. *p* values < 0.05 were considered statistically significant. All analyses were performed using SigmaStat 3.5 (Systat Software, Richmond, CA, USA).

## Data Availability

The data that support the findings of this study are available from the corresponding author upon reasonable request.

## Supplementary Materials

The following are available online at https://www.mdpi.com/1422-0067/22/2/694/s1.

## References

[1] Zender L., Spector M.S., Xue W., Flemming P., Cordon-Cardo C., Silke J., Fan S.-T., Luk J.M., Wigler M., Hannon G.J. (2006). Identification and validation of oncogenes in liver cancer using an integrative oncogenomic approach. Cell.

[2] Dong J., Feldmann G., Huang J., Wu S., Zhang N., Comerford S.A., Gayyed M.F., Anders R.A., Maitra A., Pan D. (2007). Elucidation of a universal size-control mechanism in drosophila and mammals. Cell.

[3] Zhao B., Wei X., Li W., Udan R.S., Yang Q., Kim J., Xie J., Ikenoue T., Yu J., Li L. (2007). Inactivation of YAP oncoprotein by the Hippo pathway is involved in cell contact inhibition and tissue growth control. Genes Dev..

[4] Steinhardt A.A., Gayyed M.F., Klein A.P., Dong J., Maitra A., Pan D., Montgomery E.A., Anders R.A. (2008). Expression of Yes-associated protein in common solid tumors. Hum. Pathol..

[5] Overholtzer M., Zhang J., Smolen G.A., Muir B., Li W., Sgroi D.C., Deng C.-X., Brugge J.S., Haber D.A. (2006). Transforming properties of YAP, a candidate oncogene on the chromosome 11q22 amplicon. Proc. Natl. Acad. Sci. USA.

[6] Zhao B., Kim J., Ye X., Lai Z.-C., Guan K.-L. (2009). Both TEAD-binding and WW domains are required for the growth stimulation and oncogenic transformation activity of Yes-Associated protein. Cancer Res..

[7] Zhu L., Ma G., Liu J., Deng Y., Wu Q., Chen W., Zhou Q. (2019). Prognostic significance of nuclear Yes-associated protein 1 in patients with nonsmall cell lung cancer. Medicine.

[8] Wu Y., Hou Y., Xu P., Deng Y., Liu K., Wang M., Tian T., Dai C., Li N., Hao Q. (2019). The prognostic value of YAP1 on clinical outcomes in human cancers. Aging.

[9] Wojciechowska J., Krajewski W., Bolanowski M., Kręcicki T., Zatoński T. (2016). Diabetes and cancer: A review of current knowledge. Exp. Clin. Endocrinol. Diabetes.

[10] Ryu T.Y., Park J., Scherer P.E. (2014). Hyperglycemia as a risk factor for cancer progression. Diabetes Metab. J..

[11] Zhang X., Qiao Y., Wu Q., Chen Y., Zou S., Liu X., Zhu G., Zhao Y., Chen Y., Yu Y. (2017). The essential role of YAP O-GlcNAcylation in high-glucose-stimulated liver tumorigenesis. Nat. Commun..

[12] Wang C., Jeong K., Jiang H., Guo W., Gu C., Lu Y., Liang J. (2016). YAP/TAZ regulates the insulin signaling via IRS1/2 in endometrial cancer. Am. J. Cancer Res..

[13] Chen J., Harris R.C. (2015). Interaction of the EGF receptor and the Hippo pathway in the diabetic kidney. J. Am. Soc. Nephrol. JASN.

[14] Chen C., Yang H.-I., Yang W., Liu C., Chen P., You S., Wang L., Sun C.-A., Lu S.-N., Chen D. (2008). Metabolic factors and risk of hepatocellular carcinoma by chronic hepatitis B/C infection: A follow-up study in Taiwan. Gastroenterology.

[15] Ma R., Ren J.-M., Li P., Zhou Y.-J., Zhou M.-K., Hu Z., Xiao X.-Y. (2019). Activated YAP causes renal damage of type 2 diabetic nephropathy. Eur. Rev. Med. Pharmacol. Sci..

[16] Guo X., Sun Y., Azad T., van Rensburg H.J.J., Luo J., Yang S., Liu P., Lv Z., Zhan M., Lu L. (2020). Rox8 promotes microRNA-dependent *yki* messenger RNA decay. Proc. Natl. Acad. Sci. USA.

[17] Birney E., Kumar S., Krainer A.R. (1993). Analysis of the RNA-recognition motif and RS and RGG domains: Conservation in metazoan pre-mRNA splicing factors. Nucleic Acids Res..

[18] Göhring F., Schwab B.L., Nicotera P., Leist M., Fackelmayer F.O. (1997). The novel SAR-binding domain of scaffold attachment factor A (SAF-A) is a target in apoptotic nuclear breakdown. EMBO J..

[19] Dominguez C., Fisette J.-F., Chabot B., Allain F.H.-T. (2010). Structural basis of G-tract recognition and encaging by hnRNP F quasi-RRMs. Nat. Struct. Mol. Biol..

[20] Garneau D., Revil T., Fisette J.-F., Chabot B. (2005). Heterogeneous nuclear ribonucleoprotein F/H proteins modulate the alternative splicing of the apoptotic mediator Bcl-x. J. Biol. Chem..

[21] Xu C., Xie N., Su Y., Sun Z., Liang Y., Zhang N., Liu D., Jia S., Xing X., Han L. (2019). HnRNP F/H associate with hTERC and telomerase holoenzyme to modulate telomerase function and promote cell proliferation. Cell Death Differ..

[22] Lo C.-S., Shi Y., Chang S.-Y., Abdo S., Chenier I., Filep J.G., Ingelfinger J.R., Zhang S.-L., Chan J.S. (2015). Overexpression of heterogeneous nuclear ribonucleoprotein F stimulates renal *Ace-2* gene expression and prevents TGF-β1-induced kidney injury in a mouse model of diabetes. Diabetologia.

[23] Lo C.-S., Miyata K.N., Zhao S., Ghosh A., Chang S.-Y., Chenier I., Filep J.G., Ingelfinger J.R., Zhang S.-L., Chan J.S. (2019). Tubular deficiency of heterogeneous nuclear ribonucleoprotein F elevates systolic blood pressure and induces glycosuria in mice. Sci. Rep..

[24] Lo C.-S., Shi Y., Chenier I., Ghosh A., Wu C.-H., Cailhier J.-F., Ethier J., Lattouf J.-B., Filep J.G., Ingelfinger J.R. (2017). Heterogeneous nuclear ribonucleoprotein F stimulates sirtuin-1 gene expression and attenuates nephropathy progression in diabetic mice. Diabetes.

[25] Chu W.-K., Hung L.-M., Hou C.-W., Chen J.-K. (2019). Heterogeneous ribonucleoprotein F regulates YAP expression via a G-tract in 3′UTR. Biochim. Biophys. Acta Gene Regul. Mech..

[26] Conne B., Stutz A., Vassalli J.-D. (2000). The 3′ untranslated region of messenger RNA: A molecular ‘hotspot’ for pathology?. Nat. Med..

[27] Sayed-Ahmed N., Besbas N., Mundy J., Muchaneta-Kubara E., Cope G., Pearson C., El Nahas M. (1996). Upregulation of epidermal growth factor and its receptor in the kidneys of rats with streptozotocin-induced diabetes. Exp. Nephrol..

[28] Saad S., Stevens V.A., Wassef L., Poronnik P., Kelly D.J., Gilbert R.E., Pollock C.A. (2005). High glucose transactivates the EGF receptor and up-regulates serum glucocorticoid kinase in the proximal tubule. Kidney Int..

[29] Szeto S.G., Narimatsu M., Lu M., He X., Sidiqi A.M., Tolosa M.F., Chan L., De Freitas K., Bialik J.F., Majumder S. (2016). YAP/TAZ Are mechanoregulators of TGF-β-smad signaling and renal fibrogenesis. J. Am. Soc. Nephrol. JASN.

[30] Liang M., Yu M., Xia R., Song K., Wang J., Luo J., Chen G., Cheng J. (2017). Yap/Taz deletion in Gli^+^ cell-derived myofibroblasts attenuates fibrosis. J. Am. Soc. Nephrol. JASN.

[31] Connolly S.B., Sadlier D., Kieran N.E., Doran P.P., Brady H.R. (2003). Transcriptome profiling and the pathogenesis of diabetic complications. J. Am. Soc. Nephrol. JASN.

[32] Mauger D.M., Lin C., Garcia-Blanco M.A. (2008). hnRNP H and hnRNP F complex with Fox2 to silence fibroblast growth factor receptor 2 exon IIIc. Mol. Cell. Biol..

[33] Baltz A.G., Munschauer M., Schwanhäusser B., Vasile A., Murakawa Y., Schueler M., Youngs N., Penfold-Brown D., Drew K., Milek M. (2012). The mRNA-bound proteome and its global occupancy profile on protein-coding transcripts. Mol. Cell.

