# Supplementary material for: PKC Regulates YAP Expression through Alternative Splicing of YAP 3′UTR Pre-mRNA by hnRNP F
Source: Int J Mol Sci. 2021 Jan 12;22(2):694. doi: 10.3390/ijms22020694 (PMC7828143; doi:10.3390/ijms22020694)
Supplement: Supplementary file 1 [file ijms-22-00694-s001.zip › Supplementary Table S1.docx]

**Supplementary Table 1A. List of primers used in this study**

Primer sequences for RT-PCR

| **Primer** | **Target** | **Sequence (5′-3′)^a^** |
| --- | --- | --- |
| Primer 3′UTR-F  Primer 3′UTR-R  Primer FL  Primer AS | YAP 3′UTR | F:AGCCCTCAGGCAGACTGAATTCT  R:AAGAGTTTAAGGAAAGAATATATTTG  F:CCCAACAAAACAGAACAAAATGCAC  R:CAGAAAACAATTGTTATGATAGGCACACCCACAAAA |
| YAP-F  YAP-R | YAP | F:TAGCCCTGCGTAGCCAGTTA  R:TAGTATCACCTGTATCCATCTC |
| β-actin-F  β-actin-R | β-actin | F: GCGAGAAGATGACCCAGATCATGTT  R:GCTTCTCCTTAATGTCACGCACGAT |

Primer sequences for real-time RT-PCR

| **Primer** | **Target** | **Sequence (5′-3′)^a^** |
| --- | --- | --- |
| YAP-qF  YAP-qR | YAP | F:CGCTCTTCAACGCCGTCA  R:AGTACTGGCCTGTCGGGAGT |
| β-actin-qF  β-actin-qR | β2-microglobulin | F:GCCGTGTGAACCATGTGACTTT  R:CCAAATGCGGCATCTTCAAA |

Primer sequences for cloning

| **Primer** | **Target** | **Sequence (5′-3′)^a^** |
| --- | --- | --- |
| YAP1-3UTR-F (XbaI)  YAP1-3UTR-R (FseI): | YAP3'UTR | F: cccTCTAGAagccctcaggcagactgaattct  R: cccGGCCGGCCaagagtttaaggaaagaatatatttg |

^a^Forward (F) and reverse (R) primers are indicated.

**Supplementary Table 1B. siRNA sequences**

| **siRNA** | **Sequence** |
| --- | --- |
| HnRNP F siRNA | 5'-GGUGUCCAUUUCAUCUACA-3' |
| HnRNP U siRNA | 5'-GAUGAACACUUCGAUGACA-3' |
| Control non-silencing siRNA | 5'-UUCUCCGAACGUGUCACGU-3' |
