# Supplementary material for: PKC Regulates YAP Expression through Alternative Splicing of YAP 3′UTR Pre-mRNA by hnRNP F
Source: Int J Mol Sci. 2021 Jan 12;22(2):694. doi: 10.3390/ijms22020694 (PMC7828143; doi:10.3390/ijms22020694)
Supplement: Supplementary file 1 [file ijms-22-00694-s001.zip › Data S1.pdf]

```
CLUSTAL O(1.2.4) multiple sequence alignment


G-tract      ------------------------------------------------------------     0
YAP          AGCCCTCAGGCAGACTGAATTCTAAATCTGTGAAGGATCTAAGGAGACACATGCACCGGA     60
TAZ          ------------------------------------------------------------     0
MST1         ------------------------------------------------------------     0
LATS1        ------------------------------------------------------------     0
LATS2        --------------------------------atgggggccaggcacccccaccactcgc     28


G-tract      ------------------------------------------------------------     0
YAP          AATTTCCATAAGCCAGTTGCAGTTTTCAGGCTAATACAGAAAAAGATGAACAAACGTCCA     120
TAZ          ------------------------------------------------------------     0
MST1         ------------------------------------------------------------     0
LATS1        ------------------------------------------------------------     0
LATS2        tgcctcccaggtcagggtcccg-----gagccggtgccctcacaggccaa---tagggaa     80


G-tract      ------------------------------------------------------------     0
YAP          GCAAGATACTTTAATCCTCTATTTTGCTCTTCCTTGTCCATTGCTGCTGTTAATGTATTG     180
TAZ          ------------------------------------------------------------     0
MST1         ------------------------------------------------------------     0
LATS1        ------------------------------------------------------------     0
LATS2        gccgagggctgt-----tttgttttaaattagtccgtcgattacttcacttgaaattctg     135


G-tract      ------------------------------------------------------------     0
YAP          CTGACCTCTTTCACAGTTGGCTCTAAAGAATCAAAAGAAAAAAACTTTTTATTTCTTTTG     240
TAZ          ------------------------------------------------------------     0
MST1         ------------------------------------------------------------     0
LATS1        ---------------------------------------------------------tat     3
LATS2        ctcttcaccaagaaaacccaaacaggacacttttgaaaacaggactcagcatcgct--t-     192


G-tract      ------------------------------------------------------------     0
YAP          CTATTAAAACTACTGTTCATTTTGGGGGCTGGGGGAAGTGAGCCTGTTTGGATGATGGAT     300
TAZ          ------------------------------------------------------------     0
MST1         ------------------------------------------------------------     0
LATS1        atgtttaacacactagtaa-----------------------------------------     22
LATS2        ----tcaataggcttttcaggaccttcactgcattaaaacaatatttttgaaaatttagt     248


G-tract      ------------------------------------------------------------     0
YAP          GCCATTCCTTTTGCCCAGTTAAATGT----TCACCAATCATTTTAACTAAATACTCAGAC     356
TAZ          ------------------------------------------------------------     0
MST1         ------------------------------------------------------------     0
LATS1        ------------------------------------------------------------     22
LATS2        acagtttagaaagagcacttattttgtttatatccatttttttcttactaaattatagga     308


G-tract      ------------------------------------------------------------     0
YAP          TTAGAAGTCAGATGCTTCATGTCACAGCATTTAGTTTGTTCAACAGTTGTTTCTTCAGCT     416
TAZ          ------------------------------------------------------------     0
MST1         ------------------------------------------------------------     0
LATS1        ------------------------------------------------------------     22
LATS2        ttaactttg--a---caaatcatgctgctgttattttctacatttgtattttatccatag     363


G-tract      ------------------------------------------------------------     0
YAP          TCCTTTGTCCAGTGGAAAAACATGATTTACTGGTCTGACAAGCCAAAAATGTTATATCTG     476
TAZ          ------------------------------------------------------------     0
MST1         ------------------------------------------------------------     0
LATS1        ------------------------------------------------------------     22
LATS2        cactt-------------------attcacatttaggaaaagacataaaaactgaagaac     404


G-tract      ------------------------------------------------------------     0
```

```
YAP         ATATT---AAATACTTAATGCTGATTTGAAGAGATAGCTGAAACCAAGGCTGAAGACTGT      533
TAZ         ------------------------------------------------------------      0
MST1        ------------------------------------------------------------      0
LATS1       --------ataaatgtaatgaggatttgtaaaagggcctgaa------------------      56
LATS2       attgatgagaaatctctgtgcaataatgtaaaaaaaaaaaaagataacactctgctcaat      464


G-tract     ------------------------------------------------------------      0
YAP         TTTACTTTCAGTATTTTCTTTTCCTCCTAGTGCTATCATTAGTCACATAATGACCTTGAT      593
TAZ         ------------------------------------------------------------      0
MST1        ------------------------------------------------------------      0
LATS1       ------------------------------------------------------------      56
LATS2       gtca-----------------------cggagaccattttatccacacaatggtttttgt      501


G-tract     ------------------------------------------------------------      0
YAP         TTTATTTTAGGAGCTTATAAGGCATGAGACAATTTCCATATAAATATATTAATTATTGCC      653
TAZ         ------------------------------------------------------------      0
MST1        ------------------------------------------------------------      0
LATS1       ------------------------------------------------------------      56
LATS2       tt------tttattttttcccatgtttcaaaattgtgatataatgat-------------      542


G-tract     --------------------------------TTGTGGGTGTGC----------------      12
YAP         ACATACTCTAATATAGATTTTGGTGGATAATTTTGTGGGTGTGCATTTTGTTCTGTTTTG      713
TAZ         ------------------------------------------------------------      0
MST1        ------------------------------------------------------------      0
LATS1       ---------------------------------------------------------atg      59
LATS2       --------------------------ataatgtt-------------aaaagctgctttt      563


G-tract     ------------------------------------------------------------      12
YAP         TTGGGTTTTTTGTTTTTTTTG--TTTTTGGCAGGGTCGGTGGGGGGGTTGGTTGGTTGGT      771
TAZ         ------------------------------------------------------------      0
MST1        ------------------------------------------------------------      0
LATS1       cgaggtgtttt--------------------------------------------gaggt      75
LATS2       tttggctttttgcatatctagtataataggaagtgtgagc----aaggtgatgatgtggc      619


G-tract     ------------------------------------------------------------      12
YAP         TGGTTTTGTCGGAACCTAGGCAAATGACCATATTAGTGAATCTGTTAATAGTTGTAGCTT      831
TAZ         ------------------------------------------------------------      0
MST1        ------------------------------------------------------------      0
LATS1       t--------ctgagagtaaaattatgcaaatatgacagagct---atatatgtgtgc---      121
LATS2       tgtgatttccgacgtctggtgtgt---------------------ggagagtactgcatg      658


G-tract     ------------------------------------------------------------      12
YAP         GGGATGGTTATTGTAGTTGTTTTGGTAAAATCTTCATTTCCTGGTTTTTTTTACCACCTT      891
TAZ         ------------------------------------------------------------      0
MST1        ------------------------------------------------------------      0
LATS1       -----------------tctg---------------------------------------      125
LATS2       agcagagttct------tcta-ttataaaattaccatatcttgcc-attcacagcaggtc      710


G-tract     ------------------------------------------------------------      12
YAP         ATTTAAATCTCGATTATCTGCTCTCTCTTTTATATACATACACACACCCAAACATAACAT      951
TAZ         ------------------------------------------------------------      0
MST1        ------------------------------------------------------------      0
LATS1       ------------------------------------------------------------      125
LATS2       ctgtgaatacgtttttac-tgagtgtctt-----taaatgaggtgttctagacagtgtgc      764


G-tract     ------------------------------------------------------------      12
YAP         TTATAATAGTGTGGTAGTGGAATGTATCCTTTTTTAGGTTTCCCTGCTTTCCAGTTAATT      1011
TAZ         ------------------------------------------------------------      0
MST1        ------------------------------------------------------------      0
LATS1       ------------------------------------------------------------      125
LATS2       tgat---aatgtattgtgcgggtgacctcttcgctatgattgtatctcttactgttttgt      821
```

```
G-tract      ------------------------------------------------------------      12
YAP          TTTAAAATGGTAGCGCTTTGTATGCATTTAGAATACATGACTAGTAGTTTATATTTCACT      1071
TAZ          ------------------------------------------------------------      0
MST1         ------------------------------------------------------------      0
LATS1        ------------------------------------------------------------      125
LATS2        taaagaaatgcag--atgt-------------gtaactgagaagtgatttgtgtgtgtgt      866

G-tract      ------------------------------------------------------------      12
YAP          GGTAGTT------TAAATCTG---GTTGGGGCAGTCTGCAGATGTTTGAAGTAGTTTAGT      1122
TAZ          ------------------------------------------------------------      0
MST1         ------------------------------------------------------------      0
LATS1        ------------------------------------------------------------      125
LATS2        cttggttgtgattggattctttggggggggggaactgaaacatttgtcatatactgaact      926

G-tract      ------------------------------------------------------------      12
YAP          GTTCTAGA--AAGAGCTATTACTGTGGATAGTGCCTAGGGGAGTGCTCCA--------CG      1172
TAZ          ------------------------------------------------------------      0
MST1         ---------------------------gcaaggccagg-----ctgtgagggccccagct      28
LATS1        ------------------------------------------------------------      125
LATS2        tatatacatcaaaagggattaatacagcga-tgccaaaaagtttaatcacggacacatgt      985

G-tract      ------------------------------------------------------------      12
YAP          CCCTCTGGGCATACGGTAGATATTATCTGATGAATTGGAAAGGAGCA--AACCAGAA---      1227
TAZ          ------------------------------------------------------------      0
MST1         ccacccaggctttgggtgaattctggatg---gcttgctcatgtttgttagccagcacct      85
LATS1        ------------------------------------------------------------      125
LATS2        ccgtttctgtagtccgta--tgctctttcattcttggtagagct-gg--tatgtgga---      1037

G-tract      ------------------------------------------------------------      12
YAP          ATGGCTTTATTTTCTCCCTTGGACTAAT------------------------TTTTAAGT      1263
TAZ          --------------------tcactacc--------------------------------      8
MST1         tctgctctgtcgtctctccacagcacct--t---------------------tgtgaact      122
LATS1        ------------------------------------------------------------      125
LATS2        atgccatacctctgaccctactacttaccttttttactgacagactgcccacactgaaagc      1097

G-tract      ------------------------------------------------------------      12
YAP          CTCGATTGGAATTCAGTGAGTAGGTTCATAATGTGCATGACAGAAATAAGCT--TTATAG      1321
TAZ          ------------------------------------------------------------      8
MST1         caggaatgtgcgccagtgggaagggctctcttgaca---------gtcagcgtgccatct      173
LATS1        ------------------------------------------------------------      125
LATS2        ttcagtgaatgttcttagtcctgttttcttctgttactgtcaggaaactgagtgatctaa      1157

G-tract      ------------------------------------------------------------      12
YAP          TGGTTTACCTTCATTTAGCTTTGGAAGTTTTCTTTGCCTTAGTTTTGGAAGTAAATTCTA      1381
TAZ          ------------------------------------------------------------      8
MST1         tga----------------tgtgt------------------------------------      181
LATS1        ------------------tgtacaatattttattttcctaaattatgggaaatcctttta      167
LATS2        tggttctc--tcacttttttttttgttcttttagt----------------gtactttgaa      1199

G-tract      ------------------------------------------------------------      12
YAP          GTTTGTAGTTCTCATTTGTAATGAACACATTAACGACTAGATTAAAATATTGCCTTCAAG      1441
TAZ          ------------------------------------------------------------      8
MST1         ------------------------------------------------------------      181
LATS1        a-----------------------------------------------------------      168
LATS2        gtatcaaatcttaa----------------------cttggtttaaacaatac-------      1230

G-tract      ------------------------------------------------------------      12
YAP          ATTGTTCTTACTTACAAGACTTGCTCCTACTTCTATGCTGAAAATTGACCCTGGATAGAA      1501
TAZ          ------------------------------------------------------------      8
```

```
MST1        ------------------------------------------------------------    181
LATS1       ------------------------------------------------------------    168
LATS2       ----atattcctaacc-tttgtaaaaaagcaaagattcttcaaaatgacattgaaataaa    1285

G-tract     ------------------------------------------------------------    12
YAP         TACTATAAGGTTTTGAGTTAGCTGGAAAAGTGATCAGATTAATAAATGTATATTGGTAGT    1561
TAZ         ------------------------------------------------------------    8
MST1        ------------------------------------------------------------    181
LATS1       ------------------------------------------------------------    168
LATS2       aagtaagccatacgt------------atttcttagaagtatagatgtatgtgcgtgta    1333

G-tract     ------------------------------------------------------------    12
YAP         TGAATT---TAGCAAAGAAATAGAGATAATCATGATTATACCTTTATTTTTACAGGAAGA    1618
TAZ         ------------------------------------------------------------    8
MST1        ------------------------------------------------------------    181
LATS1       ------------------------------------------------------------    168
LATS2       tacacacacacacacacacacagagataaacacaatattccttatttcaaattagtatga    1393

G-tract     ------------------------------------------------------------    12
YAP         GATGATGTAACTAGAGTATGTGTCTACAGGAGTAATAATGGTTTCCAAAGAGTATTTTTT    1678
TAZ         ------------------------------------------------------------    8
MST1        ------------------------------------------------------------    181
LATS1       ------------------------------------------------------------    168
LATS2       -ttcc--tatttaaag-tgatttatatttgagtaaaaagttcaattctttttttgcttttt    1449

G-tract     ------------------------------------------------------------    12
YAP         AAAGGAACAAAACGAGCATGAATTA------ACTCTTCAATATAA---GCTATGAAGT-A    1728
TAZ         ------------------------------------------------------------    8
MST1        ------------------------------------------------------------    181
LATS1       ------------------------------------------------------------    168
LATS2       aaaaaatctgatgcttcataattttcattatattattccacatatttttccttgaagttc    1509

G-tract     ------------------------------------------------------------    12
YAP         ATAGT------TGGTTGTGAATTAAAGTGGCACCAGCTAGCACCTCTGTGTTTTAAGGGT    1782
TAZ         ------------------------------------------------------------    8
MST1        ------------------------------------------------------------    181
LATS1       ------------------------------------------------------------    168
LATS2       ttagcataatgtatccattacttagtatatatctaggcaacaacac------ttagaagt    1563

G-tract     ------------------------------------------------------------    12
YAP         CTTTCAATGTTTCTAGAATAAGCCCTTATTTTCAAGGGTTCATAACAGGCATAAAATCTC    1842
TAZ         ------------------------------------------------------------    8
MST1        ------------------------------------------------------------    181
LATS1       ------------------------------------------------------------    168
LATS2       ttatcagtgtttaaactaaaaaaataaagattcctgtgta------ctggtttacatttg    1617

G-tract     ------------------------------------------------------------    12
YAP         TTCTCCTGGCAAAAGCTGCTATGAAAAGCCTCAGCTTGGGAAGATAGATTTTTTTCCCCC    1902
TAZ         ------------------------------------------------------------    8
MST1        ------------------------------------------------------------    181
LATS1       ------------------------------------------------------------    168
LATS2       tgtgagtggcatactcaagtctgctgtgcctgtcgtcgtga-------------------    1658

G-tract     ------------------------------------------------------------    12
YAP         CAATTACAAAATCTAAGTATTTTGGCCCTTCAATTTGGAGGAGGGCAAAAGTTGGAAGTA    1962
TAZ         ------------------------------------------------------------    8
MST1        ------------------------------------------------------------    181
LATS1       -------aatgttaatttattccagccgtttaaat-------------------------    196
LATS2       ----------ctgtcagtattctcgctatttt----atagtcgtgcca-tgttg------    1697
```

```
G-tract      ------------------------------------------------------------      12
YAP          AGAAGTTTTATTTTAAGTACTTTCAGTGCTCAAAAAAATGCAATCACTGTGTTGTATATA      2022
TAZ          ------------------------------------------------------------      8
MST1         ----------------gtatgtacattggtca----------------------------      197
LATS1        --------------------------------------------cagtatttagaaaaaa      212
LATS2        ----------------ttactcacagcgctct------------gacatactttcatgtg      1729

G-tract      ------------------------------------------------------------      12
YAP          ATAGTTCATAGGTTGATCACTCATAATAATTGACTCTAAGGCTTTTATTAAGAAAACAGC      2082
TAZ          ------------------------------------------------------------      8
MST1         -----------------------ggtatattatctcaaag--------------------      214
LATS1        at----------------------------------------tgttataaggaaagtaaa      232
LATS2        gtag-----------------------------------gttctttctcaggaactcagt      1754

G-tract      ------------------------------------------------------------      12
YAP          AGAAAGATTAAATCTTGAATTAAGTCTGGGGGGAAATGGCCACTGCAGATGGAGTTTTAG      2142
TAZ          ------------------------------------------------------------      8
MST1         ------------------------------------------------------------      214
LATS1        ttatgaactg--------------------------------------------------      242
LATS2        ttaactattatttattgatatatcattacctttgaaaagcttctactggcacaattta--      1812

G-tract      ------------------------------------------------------------      12
YAP          AGTAGTAATGAAATTCTACCTAGAATGCAAAATTGGGTATATGAATTACATAGCATGTTG      2202
TAZ          ------------------------attgtaacttggatgtagccatgaccttacatttcc      44
MST1         ------------------------------------------------------------      214
LATS1        -------------------------------------------aatattatagtcagttc      259
LATS2        ------------------------------------------------------------      1812

G-tract      ------------------------------------------------------------      12
YAP          TTGGGATTTTTTTTAATGTGCAGAAGATCAAAGCTACTTGGAAGGAGTGCCTATAATTTG      2262
TAZ          t-----------------------------------------------------------      45
MST1         ------------------------------------------------------------      214
LATS1        ttggtact----------------------------------------------------      267
LATS2        ------------------------------------------------------------      1812

G-tract      ------------------------------------------------------------      12
YAP          CCAGTAGCCACAGATTAAGATTATATCTTATATATCAGCAGATTAGCTTTAGCTTAGGGG      2322
TAZ          ------------------------------------------------------------      45
MST1         ------------------------------------------------------------      214
LATS1        ------------------------------------------------------------      267
LATS2        ------------------------------------------------------------      1812

G-tract      ------------------------------------------------------------      12
YAP          GAGGGTGGGAAAGTTTGGGGGGGGGGTTGTGAAGATTTAGGGGGACCTTGATAGAGAACT      2382
TAZ          ------------------------------------------------------------      45
MST1         ------------------------------------------------------------      214
LATS1        ------------------------------------------------------------      267
LATS2        ------------------------------------------------------------      1812

G-tract      ------------------------------------------------------------      12
YAP          TTATAAACTTCTTTCTCTTTAATAAAGACTTGTCTTACACCGTGCTGCCATTAAAGGCAG      2442
TAZ          ------------------------------------------------------------      45
MST1         ------------------------------------------------------------      214
LATS1        ------------------------------------------------------------      267
LATS2        ------------------------------------------------------------      1812

G-tract      ------------------------------------------------------------      12
YAP          CTGTTCTAGAGTTTCAGTCACCTAAGTACACCCACAAAACAATATGAATATGGAGATCTT      2502
TAZ          ------------------------------------------------------------      45
MST1         ------------------------------------------------------------      214
LATS1        ------------------------------------------------------------      267
```

```
LATS2           ------------------------------------------------------------      1812


G-tract         ------------------------------------------------------------      12
YAP             CCTTTACCCCTCAACTTTAATTTGCCCAGTTATACCTCAGTGTTGTAGCAGTACTGTGAT      2562
TAZ             ------------------------------------------------------------      45
MST1            ------------gatttatattgggcgacttttaactcagagttttaaaccccagg----      258
LATS1           ------------------------------------------------------------      267
LATS2           ------------------------------------------------------------      1812


G-tract         ------------------------------------------------------------      12
YAP             ACCTGGCACAGTGCTTTGATCTTACGATGCCCTCTGTACTGACCTGAAGGAGACCTAAGA      2622
TAZ             ------------------------------------------------------------      45
MST1            ------aacagag----------------------actcctagttgagtgatagctggga      290
LATS1           ------------------------------------------------------------      267
LATS2           ------------------------------------------------------------      1812


G-tract         ------------------------------------------------------------      12
YAP             GTCCTTTCCCTTTTTGAGTTTGAATCATAGCCTTGATGTGGTCTCTTGTTTTATGTCCTT      2682
TAZ             ------------------------------------------------------------      45
MST1            aagttttacattgt----------------------------ctg--------------t      308
LATS1           ------------------------------------------------------------      267
LATS2           ------------------------------------------------------------      1812


G-tract         ------------------------------------------------------------      12
YAP             GTTCCTAATGTAAAAGTGCTTAACTGCTTCTTGGTTGTATTGGGTAGCATTGGGATAAGA      2742
TAZ             ------------------------------------------------------------      45
MST1            ttttcttc------------------------------------------tcccaatagc      326
LATS1           ------------------------------------------------------------      267
LATS2           ------------------------------------------------------------      1812


G-tract         ------------------------------------------------------------      12
YAP             TTTTAACTGGGTATTCTTGAATTGCTTTTACAATAAACCAATTTTATAATCTTTAAATTT      2802
TAZ             ------------------------------------------------------------      45
MST1            tttcaattgttctttctggaagacttttaaaaaaatataaatatgcatat------atat      380
LATS1           ------------------------------------------------------------      267
LATS2           ------------------------------------------------------------      1812


G-tract         ------------------------------------------------------------      12
YAP             ATCAACTTTTTACATTTGTGTTATTTTCAGTCAGGGCTTCTTAGATCTACTTATGGTTGA      2862
TAZ             ---------------------------------gggcctcttggaaaaag-------tga      65
MST1            atataaat----------------------------------------------------      388
LATS1           ------------------------------------------------------------      267
LATS2           ------------------------------------------------------------      1812


G-tract         ------------------------------------------------------------      12
YAP             TGGAGCACATTGATTTGGAGTTTCAGATCTTCCAAAGCACTATTTGTTGTAATAACTTTT      2922
TAZ             tggagcagagcaagtctgcaggtgcaccacttcccgcctccatgactcgtgctccctcct      125
MST1            ------------------------------------------------------------      388
LATS1           ------------------------------------------------------------      267
LATS2           ------------------------------------------------------------      1812


G-tract         ------------------------------------------------------------      12
YAP             CTAAATGTAGTGCCTTTAAAGGAAAAATGAA-------CACAGGGAAGTGACTTTGCTAC      2975
TAZ             ttttatgttgccagtttaatcattgcctggttttgattgagagtaacttaagttaaacat      185
MST1            --------------tataaatagattcccca-------cgcaggttggtggcatctctgt      427
LATS1           ------------------------------------------------------------      267
LATS2           ------------------------------------------------------------      1812


G-tract         ------------------------------------------------------------      12
YAP             AAATAATGTTGCTGTGTTAAGTATTCATATTAAATACATGCCTTCTATATGGAACATGGC      3035
```

```
TAZ           aaataaatattctattttcattttcaaaaaaaaaaaaa--------------------aa       225
MST1          ------------------------------------------------------------       427
LATS1         ------------------------------------------------------------       267
LATS2         ------------------------------------------------------------       1812


G-tract       ------------------------------------------------------------       12
YAP           AGAAAGACTGAAAAATAACAGTAATTAATTGTGTAATTCAGAATTCATACCAATCAGTGT       3095
TAZ           aaaaaaaaaaaaaaaaaaaaaaaaaaaaaa--aaaa-----a----aaaaaaaa------       268
MST1          ------------------------------------------------------------       427
LATS1         ------------------------------------------------------------       267
LATS2         ------------------------------------------------------------       1812


G-tract       ------------------------------------------------------------       12
YAP           TGAAACTCAAACATTGCAAAAGTGGGTGGCAATATTCAGTGCTTAACACTTTTCTAGCGT       3155
TAZ           --aaaaaaaaaaaaaaaaaaa---------------------------------------       287
MST1          ------------------------------------------------------------       427
LATS1         ----------------------------------taaagtacttaaaataagtagtgctt       293
LATS2         ------------------------------------------------------------       1812


G-tract       ------------------------------------------------------------       12
YAP           TGGTACATCTGAGAAATGAGTGCTCAGGTGGATTTTATCCTCGCAAGCATGTTGTTATAA       3215
TAZ           ------------------------------------------------------------       287
MST1          ------------------------------------------------------------       427
LATS1         tgtttaaaaggagaaacctggtatctatttgtatatatgcta------------------       335
LATS2         ------------------------------------------------------------       1812


G-tract       ------------------------------------------------------------       12
YAP           GAATTGTGGGTGTGCCTATCATAACAATTGTTTTCTGTATCTTGAAAAAGTATTCTCCAC       3275
TAZ           ------------------------------------------------------------       287
MST1          ------------------------------------------------------------       427
LATS1         ------------------------------------------------------------       335
LATS2         ------------------------------------------------------------       1812


G-tract       ------------------------------------------------------------       12
YAP           ATTTTAAATGTTTTATATTAGAGAATTCTTTAATGCACACTTGTCAAATATATATATATA       3335
TAZ           ------------------------------------------------------------       287
MST1          ------------------------------------------------------------       427
LATS1         --------------------aataattttaaaatacaagagt------------------       357
LATS2         ------------------------------------------------------------       1812


G-tract       ------------------------------------------------------------       12
YAP           GTACCAATGTTACCTTTTTATTTTTTGTTTTAGATGTAAGAGCATGCTCATATGTTAGGT       3395
TAZ           ------------------------------------------------------------       287
MST1          ------------------------------------------------------------       427
LATS1         --------------ttttgaaatttttttctaga--------------------------       377
LATS2         ------------------------------------------------------------       1812


G-tract       ------------------------------------------------------------       12
YAP           ACTTACATAAATTGTTACATTATTTTTTCTTATGTAATACCTTTTTGTTTGTTTATGTGG       3455
TAZ           ------------------------------------------------------------       287
MST1          ------------------------------------------------------------       427
LATS1         ------------------------------------------------------------       377
LATS2         ------------------------------------------------------------       1812


G-tract       --------------------------------------     12
YAP           TTCAAATATATTCTTTCCTTAAACTCTTAAAAAAAAAA     3493
TAZ           --------------------------------------     287
MST1          --------------------------------------     427
LATS1         --------------------------------------     377
LATS2         ---------------ttattaaaattttgaatccaaa-     1834
```
